# Epitaxies of Ca sulfates on calcite. II. The main {010}, {001} and {100} forms of bassanite epi-deposited on the {10.4} substrate form of calcite

**DOI:** 10.1107/S1600576722008196

**Published:** 2022-09-28

**Authors:** Dino Aquilano, Marco Bruno, Stefano Ghignone, Linda Pastero, Andrea Cotellucci

**Affiliations:** a Dipartimento di Scienze della Terra, Università degli Studi di Torino, Via Valperga Caluso 35, Torino 10125, Italy; b SpectraLab s.r.l., Spin-off Accademico dell’Università degli Studi di Torino, Via G. Quarello 15/a, Torino 10135, Italy; c NIS, Centre for Nanostructured Interfaces and Surfaces, Università degli Studi di Torino, Via G. Quarello 15/a, Torino 10135, Italy; Instituto Andaluz de Ciencias de la Tierra, Granada, Spain

**Keywords:** bassanite, calcite, epitaxy, twins, anomalous mixed crystals

## Abstract

2D and 3D epitaxies of the main {010}, {001} and {100} forms of deposited bassanite (CaSO_4_·0.5H_2_O) on {10.4} calcite (CaCO_3_) as a substrate are described to provide a theoretical crystallographic background for the replacement of calcite by bassanite both in nature and in the laboratory and by weathering linked to cultural heritage. First, epitaxy in the third dimension, perpendicular to the investigated interfaces, has been verified in order to establish whether adsorption/absorption can occur (as anomalous mixed crystals) at the bassanite/calcite epi-contacts. Secondly, and by applying the Hartman–Perdok method, 2D lattice coincidences have been obtained from the physical-geometric matches of bonds running in the common directions within the elementary slices facing the substrate/deposit interfaces. This research represents the second and more detailed part of a wider program extended to the epi-interactions between the following pairs: (i) {010}-gypsum/{10.4}-calcite (just published); (ii) bassanite/{10.4}-calcite (the present work); and (iii) anhydrite (CaSO_4_)/{10.4}-calcite (coming soon).

## Abstract 2

A detailed description of 2D and 3D epitaxies of the main {010}, {001} and {100} forms of deposited bassanite (CaSO_4_·0.5H_2_O) on {10.4} calcite (CaCO_3_) is reported.

## 1. Introduction

We recently investigated all compatible 3D and 2D lattice coincidences (herein 3D- and/or 2D-LCs) that can occur at the interface between the {10.4} form of cleaved calcite (substrate) and the {010} pinacoid of gypsum (deposit) (Aquilano *et al.*, 2022 ▸). Working on calcium sulfates deposited on gypsum, we knew that when gypsum is heated above ∼150°C in the dry state (or at 75°C in methanol–water solutions), a part of the crystalline water is removed and CaSO_4_·0.5H_2_O is formed (Maslyk *et al.*, 2022 ▸). This mineral, metastable at all temperatures, occurs in nature as bassanite (Bss) (Weiss & Bräu, 2009 ▸) and, as a biomineral, in some deep-sea medusae (Tiemann *et al.*, 2002 ▸; Becker *et al.*, 2005 ▸). In recent times, bassanite has been shown to play an important role in the complex system where gypsum, bassanite and anhydrite replace calcite (Cc) – in both natural and industrial processes. The authors who work in this sector have mainly focused on studies in the thermodynamic and kinetic fields, using the most advanced techniques of characterization. Thus, bassanite has been viewed as a precursor of gypsum (Van Driessche *et al.*, 2012 ▸), or as a key product among the Ca sulfates replacing gypsum (Ruiz-Agudo *et al.*, 2015 ▸, 2016 ▸). During recent years, we have arrived at the point of formulating a tentative general model for Ca sulfate precipitation from solutions and, through nucleation, to explain the occurrence of bassanite on the surface of Mars (Stawski *et al.*, 2020 ▸).

Of all this work, the study we consider the most representative was conducted by Ruiz-Agudo *et al.* (2016 ▸), who determined the 3D crystallographic relationship between calcite (parent) and Ca sulfates (products) using X-ray texture analysis. They chemically obtained all the CaSO_4_ crystalline phases from the reaction of H_2_SO_4_ foreign solutions with the replaced calcite and characterized the CaCO_3_–CaSO_4_ transformations by means of their experimental 2D X-ray diffraction analyses. Through this method, they indicated a clear crystal preferred orientation of the three Ca sulfate phases (both hydrated and anhydrous) formed during the interaction of calcite with sulfate-bearing solutions; briefly, they found that an epitaxial {10.4}_Cc_/{010}_Bss_ relationship was observed in such a way that ‘…the orientation of the parent calcite determines the disposition of the crystals of the final CaSO_4_ phase during transformation. The exact mechanism by which the crystallographic information is transferred in a dissolution–precipitation reaction is not well understood yet’ (Ruiz-Agudo *et al.*, 2016 ▸). Using our notations, the geometry of the epitaxy (in Å) was described, at that time, as follows: only [001]_Bss_ = 6.336 is parallel to the 1/3[441]_Cc_ vector = 6.425, the linear misfit between them only reaching 1.45%. No other match was found at the calcite/bassanite epi-contact, and this suggests that only 1D- and not 2D-LCs can exist at this interface, in our opinion.

Here, we did not intend to repeat experiments already carried out by others, but only to integrate them and establish a useful tool for comparison and complementary purposes; moreover, we wanted to intervene only when the rules of epitaxy have been clearly violated, especially the crystallographic ones.

Starting from this background and bearing in mind that searching for 2D epitaxy among low-symmetry structures is not always easy, we aim in the present work to investigate all compatible 2D-LCs among the {10.4}_Cc_ and the main {010}, {001}, {100} morphological forms of bassanite (Becker *et al.*, 2005 ▸; Ruiz-Agudo *et al.*, 2016 ▸). It is therefore a matter of starting from scratch, slowly moving in crystallographic morphology: to do this, we began with the surface profiles of all the involved forms, obtained through a strict application of the Hartman–Perdok method (Hartman, 1973 ▸). As we recently treated the {10.4}-calcite/{010}-gypsum epitaxy (Aquilano *et al.*, 2022 ▸) in the same way, we will complete our program in a subsequent study dealing with the {10.4}_Cc_/anhydrite (CaSO_4_) epitaxy, starting from crystallographic experience (Aquilano *et al.*, 1992 ▸) acquired many years ago.

## 2. A short summary on the {10.4} surfaces of calcite

The usual unit cell (in Å) of rhombohedral calcite (space group *R* 3/*c*) reads *a*_0_ = *b*_0_ = 4.9896; *c*_0_ = 17.06, α = β = 90°, γ = 120°. For the rectangular 2D cell of its {10.4} form, the vectors are [010] = 4.9896 and 1/3[421] = 8.103, this cleavage form being limited by a set of symmetry-equivalent vectors 1/3〈[441]〉 = 12.85, running parallel to the {10.4} edges. Each {10.4} face shows a sharp pseudohexagonal symmetry; in fact, a large supercell occupying an area of 242.58 Å^2^ with multiplicity (6×) can be drawn (Fig. S1 of the supporting information), and these features are more pronounced when we consider the epi-relationship of calcite/bassanite.

**Figure 1:**
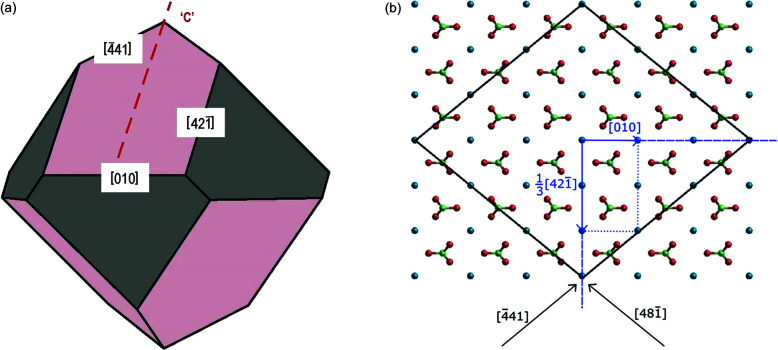
(*a*) Cleavage {10.4} calcite rhombohedron (dark-pink colour) with its PBCs running along the vertical glide plane ‘*c*’ parallel to 〈421〉 and the horizontal 〈010〉 directions; the brown parts are related to the {01.2} form. (*b*) Perpendicular view of the same rhombohedron, where the [421] and [010] directions are indicated; Ca is blue, C is green and O is red.

The {10.4}_Cc_ is a flat (F) form growing through the layer-by-layer mechanism (either 2D nucleation or spiral, or both). Actually, four periodic bond chains (PBCs) run within the slice *d*_10.4_ = 3.034 Å thick. The two main PBCs develop along the 〈441〉 and 〈481〉 directions, made equivalent through the glide plane ‘*c*’ [Fig. 1 ▸(*a*)], so building all the edges limiting the six rhombohedron faces. The two other main vectors run along the 〈421〉 and 〈010〉 directions. It is fundamental here to recollect the related PBC strength (the end chain energy, erg ion^−1^ × 10^10^), *i.e.* the energy released when an ion enters, in a crystallographic position, at one end of each semi-infinite chain: 0.391, 0.359 and 0.333 for the PBCs 〈441〉, 〈421〉 and 〈010〉, respectively (Ruiz-Agudo *et al.*, 2016 ▸; Stawski *et al.*, 2020 ▸). It has also been demonstrated that there is only one way to choose the surface profile of {10.4}. Accordingly, the {10.4} profile does not need to be reconstructed, since no atoms can be found on the ideal planes separating two adjacent *d*_10.4_ slices. In other words, *d*_10.4_ are ‘self-consistent slices’. Thirty years ago, more or less, we quantified its compactness (Hartman, 1973 ▸), *i.e.* the interaction energy ( 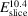 ) between the atoms contained within the *d*_10.4_ slice, and found that 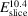 = 0.222 erg × 10^−10^ ion^−1^, corresponding to no less than ∼94% of the calcite crystallization energy (Aquilano *et al.*, 1992 ▸). The shape of growth (or dissolution) of {10.4} patterns (spirals and/or 2D nuclei) is theoretically defined (Hartman, 1973 ▸) by the 〈441〉 steps limiting the faces, followed by the 〈421〉 vertical and 〈010〉 horizontal directions, as anticipated and demonstrated in Fig. 1 ▸.

## 3. Bassanite {010}: the sharp stacking difference of its elementary *d*_020_ layers with respect to those of the {001} and {100} forms. A comparison with the *d*_10.4_ layers of the cleavage calcite rhombohedron

**Figure 2:**
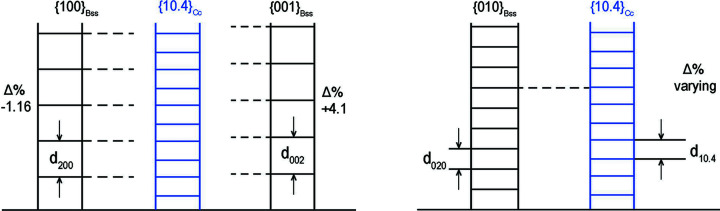
Simple drawing to illustrate, as a whole, the data described in Table 1 ▸. Misfits and coincidences between the layer thicknesses are outlined.

Table 1 ▸ and Fig. 2 ▸ show the profound differences between the behaviour of the main forms {010}, {001} and {100} of bassanite with respect to the same {10.4}_Cc_ substrate. In other words, there is no compatibility between the thickness of the elementary slices *d*_10.4_ = 3.043 Å (calcite) and *d*_020_ = 3.4635 Å (bassanite). To find an acceptable correspondence in the thickness (10.4)_Cc_/(010)_Bss_, one has to rise up to the thickness of (6–9) × *d*_020_ layers of bassanite; after this, the misfit starts to rise again (from +2.43%) and adsorption/absorption of 2D-{010}_Bss_ layers into the bulk of the {10.4} form of calcite becomes improbable in comparison with the cases for other basic bassanite forms. This means that bassanite adsorption can only occur at the (10.4)_Cc_/(010)_Bss_ interface. Fig. 2 ▸ provides evidence showing that, for the other two interfaces (10.4)_Cc_/(001)_Bss_ and (10.4)_Cc_/(100)_Bss_, the thickness correspondences are everywhere very close to each other, in such a way that the misfit reaches a maximum of −1.16 and +4.1% for *d*_200_ and *d*_002_, respectively. Underlining this difference is useful, as it highlights the pseudo-quadratic 2D symmetry of (010)_Bss_ with respect to the pseudo-hexagonality of both the (100) and the (001) planes.

### 3.1. The pseudo-quadratic hexagonal symmetry of bassanite viewed along [010] and the pseudo-hexagonal symmetry along both [100] and [001] directions

**Figure 3:**
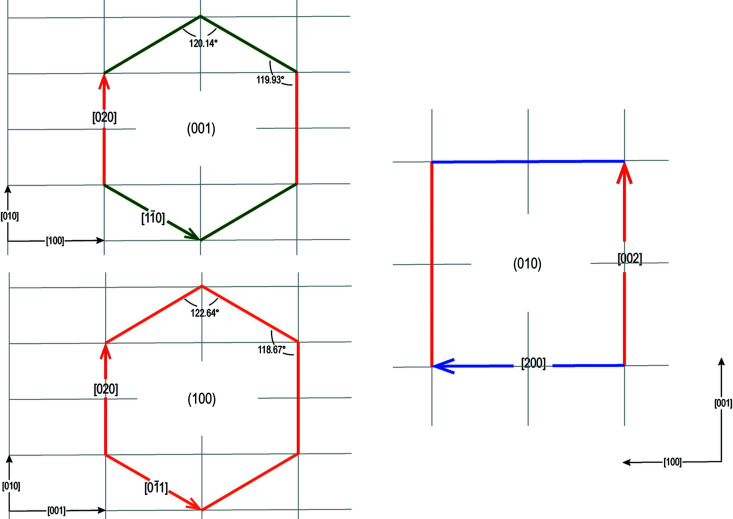
Pseudo-symmetry of bassanite forms. It is pseudo-hexagonal (left side) along the [100] and [001] directions, as described in Section 3.1. However, it appears pseudo-quadratic (right side) viewed along [010]. In this case, the supercell vectors refer to cases 4a and 4b (Table 2 ▸).

As described in the *Introduction* [Sec sec1], we adopted the bassanite structure proposed by Ballirano *et al.* (2001 ▸) and Hildyard *et al.* (2011 ▸), who determined the monoclinic space group *I*2 and the cell parameters (in Å) *a*_0_ = 12.032, *b*_0_ = 6.927, *c*_0_ = 12.671 and β = 90.27°. Fig. 3 ▸ roughly describes the sub-symmetry of bassanite:

The right side shows that the {010} form looks ‘pseudo-quadratic’; in fact, the vector [200]_Bss_ = 24.064 and its perpendicular [002]_Bss_ = 25.342 differ by a misfit of 5.31%. The comparison between the supercell made by these two vectors and that made by calcite [421] = 24.309 and 5[010] = 24.948, building the {10.4}_Cc_ supercell, points out the striking 2D-LCs (see cases 4a and 4b in Table 2 ▸) occurring between {010}_Bss_ and {10.4}_Cc_.

The left side outlines that the {001} and {100} forms are ‘pseudohexagonal’. In fact, (i) the vertical side is common and has a length of 13.854; (ii) the diagonal ones have lengths of 13.883 and 14.441 in the forms {001} and {100}, respectively; and (iii) the six internal angles range from 119.93 to 120.14° in the {001} form, and from 118.67 to 122.64° in the {100} form. The areas (in Å^2^) of these 2D-LCs [multiplicity (6×)] vary from 500.074 to 526.63, going from the {001} to {100} forms. The resulting Δ% reaches 5.31.

To summarize, through the exposed surfaces, we can obtain further proof that it is reasonable to treat {010}_Bss_ separately from {001} and {100}, when the epi-contact with {10.4}_Cc_ is made.

### 3.2. 2D coincidence lattices between bassanite {010} and calcite {10.4}

**Figure 4:**
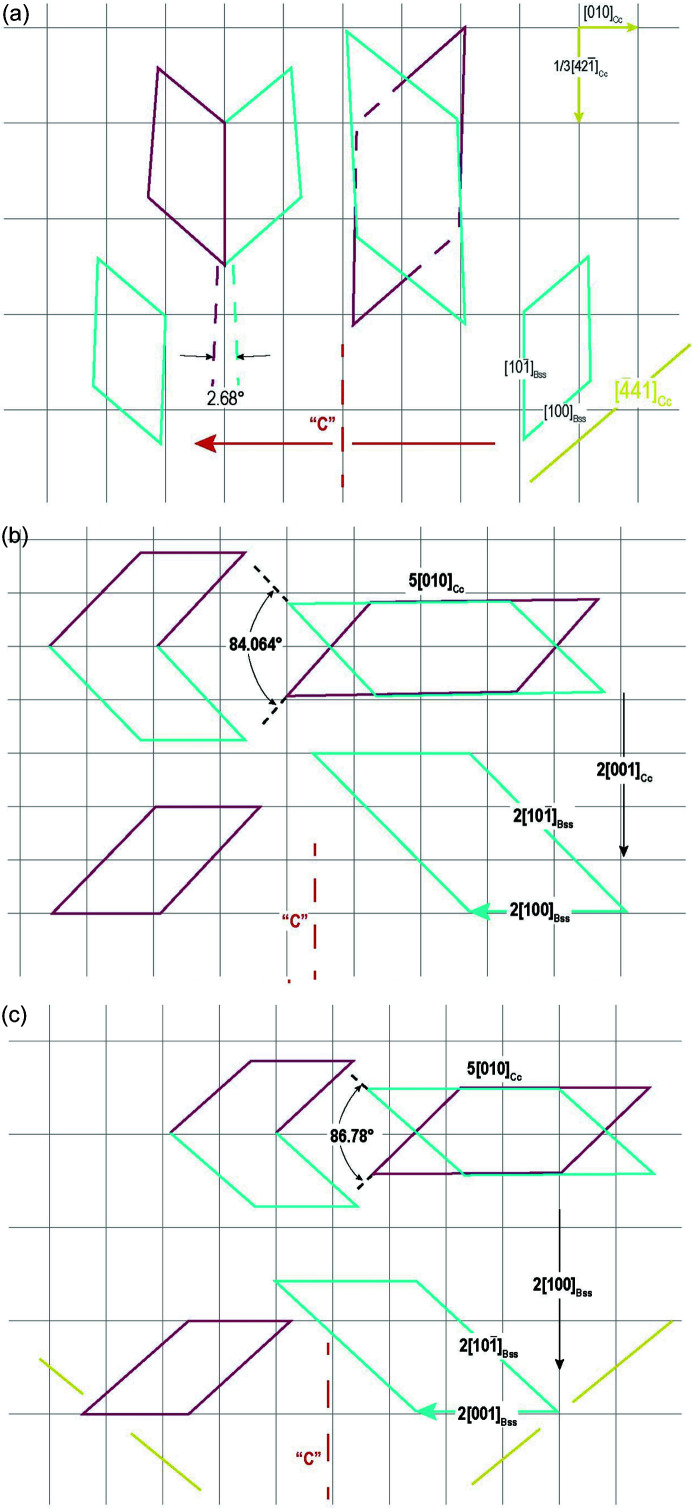
(*a*) Drawing of case 1 from Table 2 ▸. The side (blue) of the parent nucleus, [100]_Bss_, is parallel to one main side [441] of the calcite substrate, whereas the other side, [101]_Bss_, is nearly parallel to one of the other main PBCs [421]_Cc_. The angular misfit between [101]_Bss_ and [441]_Cc_ is very low at 1.34°. This means that we are dealing with a new 2D twin law generated by the {010}_Bss_/{10.4}_Cc_ epitaxy. This 2D twin law has the [101] axis of bassanite. (*b*) In case 4a (Table 2 ▸), we observe [100]_Bss_//5[010]_Cc_. (*c*) In case 4b, we obtain 2[001]_Bss_//5[010]_Cc_. Note, we can obtain two new 2D twin laws for bassanite: twin axes [100]_Bss_ and [001]_Bss_ for cases 4a and 4b, respectively. Finally, all the cases illustrated in (*b*) and (*c*) show that the linear misfits run in the opposite sense, which would be the ideal situation to calculate (010)_Bss_/(10.4)_Cc_ adhesion energy.

According to the preceding sections, the best fit between a vector in the (010) plane of bassanite and a chain in the (10.4) plane of calcite is that between the most important edge 1/3〈441〉_calcite_ = 12.85 Å and the most important axis of bassanite [001]_Bss_ = 12.671 Å, the linear misfit being 1.45%. Accordingly, the statement (Ruiz-Agudo *et al.*, 2016 ▸) that no 2D-LC can be found at this interface (see the *Introduction* [Sec sec1]) is rather pessimistic. In fact, from Table 2 ▸ and Fig. 4 ▸, one can find the following:

(i) The [441]_Cc_ chain is fundamental to build up an epitaxy on (010)_Bss_, and the corresponding side of the shared 2D-LC is [100]_Bss_. The linear misfit between the two vectors is −6.84%, which is compensated by the opposite misfit (+7.82%) occurring between the two other sides of the 2D-LC. The linear compensation is reflected in the low value (∼6%) of the maximum area misfit which, together with the minimum multiplicity (1×) of the 2D-LC of bassanite and its tolerable (4.44°) obliquity value, allows us to say that the epitaxy constraints in case 1 are well satisfied.

(ii) Another reasonable condition that could be found for a 2D epitaxy occurs with case 2 (Table 2 ▸). The epitaxy constraints are also fulfilled in this case, but the linear misfits are coherent and hence the error propagates towards the long-range interactions, and the multiplicity of the 2D common area is twice the preceding value. Accordingly, the probability of epitaxy exists, but is lowered.

(iii) In cases 3a and 3b, the 2D common areas or angular misfits (or both) exceed the geometrical constraints in order for an epitaxy to occur.

(iv) In cases 4a and 4b, one obtains the largest 2D common areas but the best of the angular misfits. Furthermore, the linear misfits are in opposition everywhere.

To summarize, {010}_Bss_ has two opportunities to form good epitaxies with {10.4}_Cc_. Case (1) illustrates the ‘short-range’ 2D-LC, owing to the lowest multiplicity (1×) of the {010}_Bss_ lattice. Cases 4a and 4b describe the ‘long-range’ 2D-LC, as it ensues from the multiplicity (4×). By now, only the values of the adhesion energy between {010}_Bss_ and {10.4}_Cc_ could indicate which one of the epitaxies will be the preferred one. Anyway, it is worth remembering that the small 2D epi-nuclei of bassanite could form at medium–high supersaturation, whereas the larger ones are stable even at low supersaturation (with respect to bassanite). In the first case, short-range 2D-LCs are coupled with small nuclei, whereas the long-range ones will be coupled with the larger nuclei. Accordingly, this is the best evidence that epitaxy {010}_Bss_/{10.4}_Cc_ has good probability to occur.

From the occurrence frequency expressed in Table 2 ▸, one obtains these observed rules:

(i) [100]_Bss_ is parallel to [441]_Cc_, [421]_Cc_, [010]_Cc_ and, less frequently, to [4.19.1]_Cc_,

(ii) [001]_Bss_ is parallel to [421]_Cc_, [010]_Cc_ and, less frequently, to [4.17.1]_Cc_,

(iii) [101]_Bss_ is parallel to [421]_Cc_ and, less frequently, to [451]_Cc_ and [4.11.1]_Cc_,

(iv) [201]_Bss_ is parallel to [441]_Cc_,

(v) [101]_Bss_ is parallel to [4.11.1]_Cc_.

In other words, one has to search for these alignments, having remembered that only adsorption of bassanite on calcite can occur, according to the last row of Table 1 ▸. In these cases, (for adsorption alone) the action of screw dislocation cannot be foreseen at the outcropping calcite/bassanite interface: this means that periodic polysynthetic twins cannot be obtained on the growing surfaces (Boistelle & Aquilano, 1977 ▸; Aquilano, 1977 ▸), although they could easily occur when adsorption/absorption mixes one or more complex interfaces.

The situation shown by 2D-LCs (cases 4a, 4b) is quite interesting. In fact, in both cases, the linear and 2D-area misfit are very low or negligible; the obliquity is nil; the linear misfits are opposite. Finally, and this is amazing, both 2D-LCs are practically quadratic, the directions of their sides being parallel to the cell axes *a*_0_ and *c*_0_ of bassanite. It is not by chance that we suppose [in Fig. 4 ▸(*b*)] the reasonable existence of 〈101〉 ledges in bassanite, to avoid the superposition of the original parent embryos.

### 3.3. The surface structures of the {001} form of bassanite

**Figure 5:**
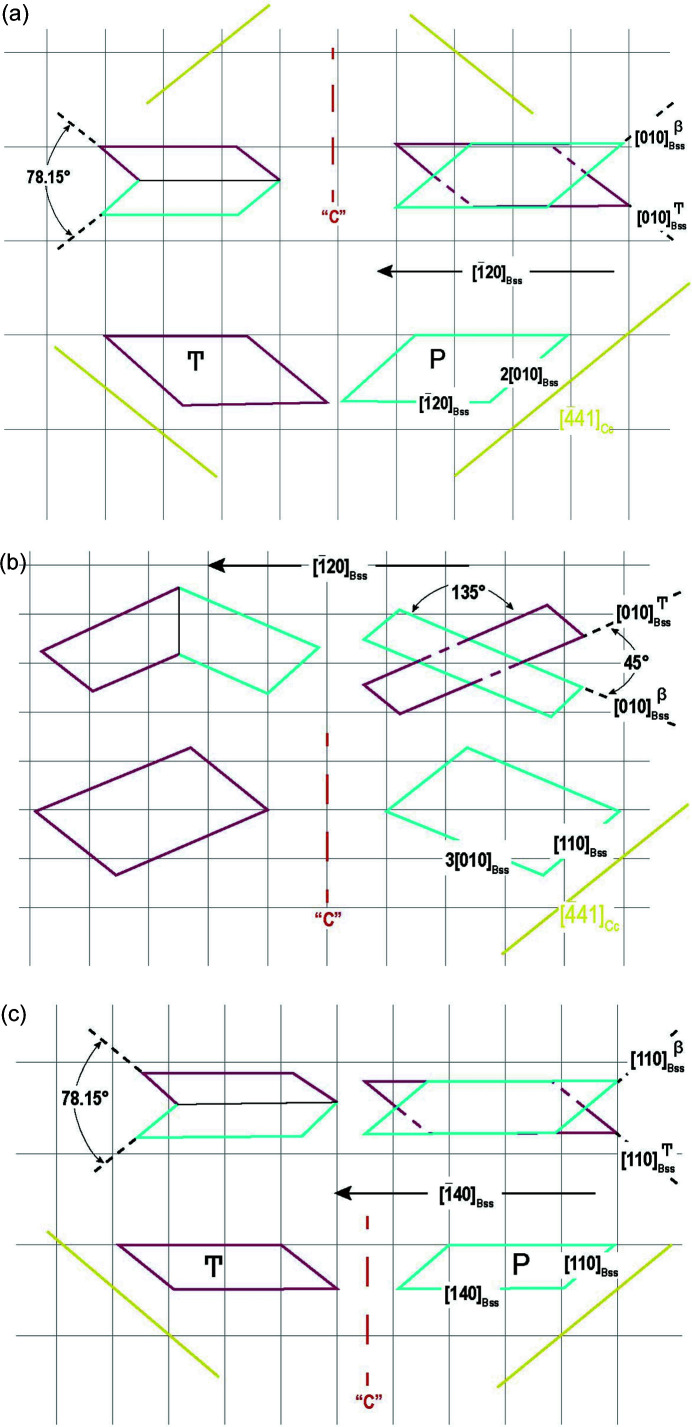
Three examples of 2D-LCs between {001}_Bss_ and {10.4}_Cc_. It is coincidental that the interpenetration twins, described in cases 1 and 2b, and drawn in (*a*) and (*c*), lead to the same swallow angle, even though they are formed by different bassanite sides in the two cases.

In cases 1, 2a and 2b described in Table 3 ▸, the sides [010]_Bss_ and [110]_Bss_ exactly coincide with the sides of the pseudohexagonal 2D supercell described in Fig. 3 ▸ (left). In Figs. 5 ▸(*a*) and 5(*b*) the 2D twin law is the same: [120] is the twin axis, even if in Fig. 5 ▸(*a*) [010]_Bss_ is parallel to [441]_Cc_, whereas in Fig. 5 ▸(*b*), [110]_Bss_//[441]_Cc_. In Fig. 5 ▸(*c*) the 2D twin law changes too: [140] is the new twin axis. Note that the angle of 78.15° is the same in the ‘swallow tail’ of different laws (upper side left and lower side), because in both cases the twin axis runs parallel to the main [010] calcite PBC.

### 3.4. The surface structures of the {100} form of bassanite

**Figure 6:**
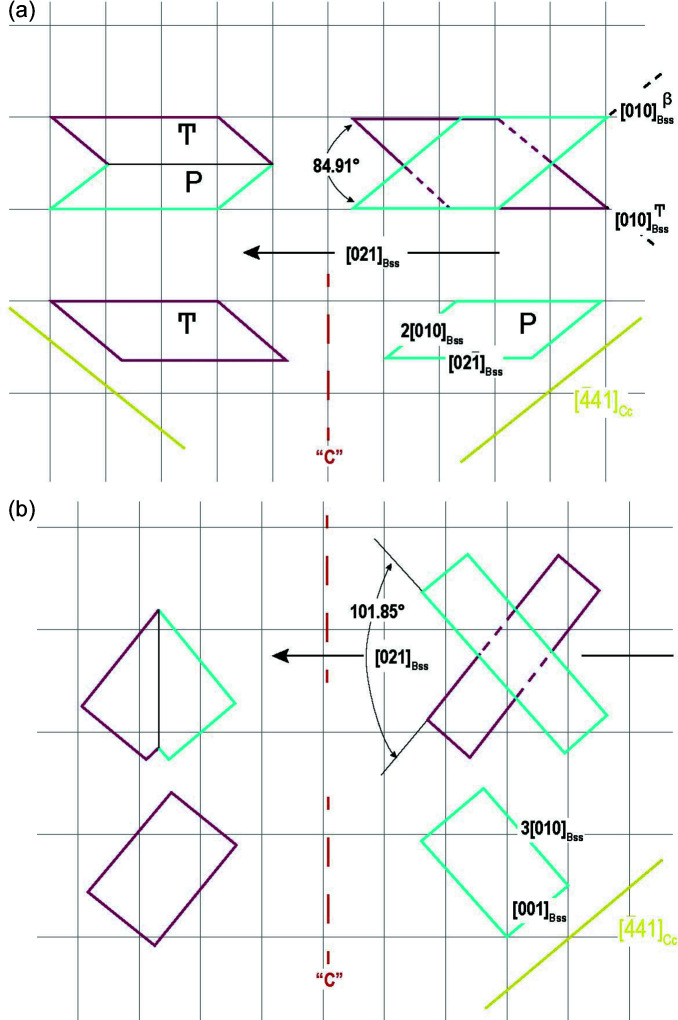
{100}_Bss_/{10.4}_Cc_epitaxy. Only the sides of the 2D-{100} cell can be found parallel to the lateral 〈441〉 sides of calcite substrate. In both cases the bassanite twin axes are parallel to [010]_Cc_, one of the main PBCs of the substrate.

In both cases of Table 4 ▸, the {100} bassanite nucleus has its sides parallel to the most important 〈441〉 PBC of the substrate. In the first case [Fig. 6 ▸(*a*)], a new 2D twin axis [021]_Bss_ is obtained. The penetration twin has a swallow angle of 84.91° determined by the 〈010〉 directions of the bassanite parent (P) and ‘*c*’ twinned (T) individuals. The lateral sides of the penetration twin are both parallel to the other sides of the nucleus and coincide with the 2D twin axis [021] of bassanite. Concerning case 2 [Fig. 6 ▸(*b*)], another 2D twin axis [032]_Bss_ works: the angle formed by the ‘*c*’ equivalent 〈010〉 bassanite directions is 101.85°.

## 4. Conclusions

Starting from the premise cited in the *Introduction* [Sec sec1], it could be assumed that bassanite is a valuable replacement for CaSO_4_ to make 2D and/or 3D epitaxy with the {1.04} cleaved form of calcite. By closely observing the interface between bassanite and {10.4}_Cc_ with deeper crystallographic insight, we instead realized in the present work that all the main bassanite forms can produce new 2D twin laws, when in epi-contact with the basic {10.4}_Cc_ rhombohedron. By taking into account the point established in Section 3 on the properties of the interfaces, we can summarize the following interactions between the calcite substrate and the new twin laws determined by bassanite deposition:

(i) {010}_Bss_: three twin laws were determined, [101]_Bss_, [100]_Bss_ and [001]_Bss_. In the first 2D-[101]_Bss_ twin law, the swallow-tail angle (92.68°) is formed by the 〈100〉_Bss_ steps and can be attributed to the ‘*c*’ glide plane that is invariably present in the {10.4} cleaved calcite. In the other two laws ([100]_Bss_ and [001]_Bss_), the swallow angle that originated between the 〈101〉 directions varies between 84.07° (2D-[100]_Bss_ twin law) and 86.78° (2D-[001]_Bss_ twin law). Readers will notice that the maximal variation in these three swallow angles is minimal (<9°) and that visually the triplets look alike, so that they can be easily confused; actually, they differ from each other both in physics and in geometry.

(ii) {001}_Bss_: first, it is remarkable that in all three main twin laws the bassanite {001} nuclei are always perfectly aligned along the 〈441〉_Cc_ sides. When the alignment is parallel to [010]_Bss_ or [110]_Bss_, a new twin law arises, with [120]_Bss_ as a new 2D twin axis (cases 1, 2a); instead, in case 2b, where [110]_Bss_ is again aligned along the 〈441〉_Cc_ side, [140]_Bss_ becomes the new 2D twin axis. Three other cases are given in Table S1 of the supporting information, either because some coincidence has been found with the 〈441〉 sides of calcite or because the 2D-Cl area misfit is too high. In two of them, the new 2D twin axes are [210]_Bss_ and [010]_Bss_. Finally, also for this bassanite form, cases 1 and 2b show the same bassanite swallow angle (81.89°), which differs only by a total obliquity of 3.74° with respect to the theoretical one (calcite), as drawn in Fig. 5 ▸ of the text.

(iii) {100}_Bss_: despite the hexagonal pseudosymmetry of bassanite, the {0*kl*} forms have fewer 2D-CL lattices than the previous ones, the weak difference between *a*_0_ and *c*_0_ of bassanite being the cause. New twins have been calculated, the new 2D twin axes being [021]_Bss_ and [032]_Bss_. In case 1, the swallow angle formed on this occasion by the two 〈010〉 equivalent directions of the two bassanite individuals reaches 84.91°. Once again, this highlights how deceptive first appearances can be when observing ‘swallow-tail twins’.

Accurate research, based on the lattice epi-correspondence between the main bassanite and (10.4)_Cc_ PBCs, allowed us to identify nine unexpected 2D twin laws, generated by the intrinsic (10.4)_Cc_ symmetry coupled with the surface symmetry of bassanite, which increasingly works like a transition compound. Further, swallow-tail twins do theoretically occur in all three cases of the epitaxies of bassanite on (10.4)_Cc_. In brief, these new 2D twin laws have been found for bassanite, promoted by the calcite substrate; together with the (10.4)_Cc_/(010)_Gypsum_ coupling examined earlier (Aquilano *et al.*, 2022 ▸), a new way of thinking is being developed in detail about the epitaxy between different species. This can be particularly useful when a new mineralogical species tends to replace another, as in the case of Ca sulfates (gypsum, bassanite, anhydrite) replacing calcite, in nature and/or the laboratory (Ruiz-Agudo *et al.*, 2016 ▸). According to our planning, the next step will be the CaSO_4_ anhydrite/(10.4)_Cc_ epitaxy.

## Supplementary Material

Supporting figure and table. DOI: 10.1107/S1600576722008196/gj5284sup1.pdf

## Figures and Tables

**Table:** The related misfits have also been shown.

	{10.4}_Cc_		{010}_Bss_		Maximum misfit (Δ%)	Notes
Layers of minimum thickness (Å)	*d*_10.4_ = 3.043		*d*_020_ = 3.4635		+13.81	–
	…and then…		…and then…		and then...	
	7*d*_10.4_ = 21.301		6*d*_020_ = 20.781		−2.5	
	8*d*_10.4_ = 24.344		7*d*_020_ = 24.2445		−0.41	
	9*d*_10.4_ = 27.382		8*d*_020_ = 27.708		+1.44	
	10*d*_10.4_ = 30.43		9*d*_020_ = 31.1715		+2.43	
					….and hence the misfit is growing again…	

**Table:** 

	{10.4}_Cc_		{001}_Bss_		Maximum misfit (Δ%)	Notes
Layers of minimum thickness (Å)	*d*_10.4_ = 3.043		*d*_002_ = 6.335		−4.1	Valid for all thicknesses of bassanite: (2*n* × *d*_002_) and calcite: (2*n* + 1)*d*_10.4_
	*2d*_10.4_ = 6.086		2*d*_002_ = 12.670		−4.1	
	4*d*_10.4_ = 12.172		and so on…		and so on…	
	and so on…					

**Table:** 

	{10.4}_Cc_		{100}_Bss_		Maximum misfit (Δ%)	Notes
Layers of minimum thickness (Å)	*d*_10.4_ = 3.043		*d*_200_ = 6.0159		−1.16	Valid for all thicknesses of bassanite: (2*n* × *d*_200_) and calcite: (2*n* + 1)*d*_10.4_
	2*d*_10.4_ = 6.086		4*d*_200_ = 12.0319		−1.16	
	4*d*_10.4_ = 12.172		and so on…		and so on…	
	and so on…					

**Table 2:** 2D-LCs between the {10.4}_Cc_ and the {010}_Bss_ pinacoid Cases 2, 3a and 3b are discussed in the supporting information as their bassanite lattice vectors are not parallel to the (10.4)_Cc_ PBCs, as in case 2. Area misfit is too large, as in cases 3a and 3b. Cases 4a and 4b, which intrinsically differ from each other, reproduce near-identical 2D-LCs.

Ranking	{10.4}_Cc_ lattice vectors (Å)	{010}_Bss_ lattice vectors (Å)	Maximum linear and area misfit (Δ%)	Obliquity (°)	Notes
Case 1	1/3[441] = 12.85	[100] = 12.032	−6.84		2D twin law axis [101]_Bss_
	−2/3[421] = 16.206	[101] = 17.5145	+7.82		
2D cell area (Å^2^) and multiplicity	161.72 (4×)	152.46 (1×)	−6.07	4.44	Opposite misfits

Case 2	1/3[4.17.1] = 26.231	2[001] = 25.342	−3.50		
	−2/3[451] = 19.137	[101] = 17.5145	−9.26		
2D cell area (Å^2^) and multiplicity	323.44 (8×)	304.91 (2×)	−6.07	3.00	Coherent misfits

Case 3a	−2/3[441] = 25.7	[201] = 27.196	+5.82		
	−1/3[4.11.1] = 17.021	−[101] = 17.5145	+2.90		
2D cell area (Å^2^) and multiplicity	404.31 (10×)	457.37 (3×)	+13.12	6.62	Coherent misfits

Case 3b	1/3[4.19.1] = 35.855	3[100] = 36.096	+0.67		
	1/3[4.11.1] = 17.021	−[101] = 17.5145	+2.90		
2D cell area (Å^2^) and multiplicity	404.31 (10×)	457.37 (3×)	+13.12	4.99	Coherent misfits

Case 4a	5[010] = 24.948	2[100] = 24.064	−3.67		2D twin law axis [100]_Bss_
	[421] = 24.309	2[001] = 25.342	+4.25		
2D cell area (Å^2^) and multiplicity	606.46 (15×)	609.83 (4×)	+0.76	0	Opposite misfits

Case 4b	[421] = 24. 309	2[100] = 24.064	−31.02		2D twin law axis [001]_Bss_
	5[010] = 24.948	2[001] = 25.342	+1.79		
2D cell area (Å^2^) and multiplicity	606.46 (15×)	609.83 (4×)	+0.76	0	Opposite misfits

**Table 3:** 2D-LCs between {001}_Bss_ and {10.4}_Cc_ The form {001}_Bss_ should have slices of thickness *d*_002_, as required by the constraint 00*l* → *l* = 2*n*. Cases 4a and 4b are provided in Table S1, as the bassanite sides are not parallel to the 〈441〉 sides of calcite

Ranking	{10.4}_Cc_ lattice vectors (Å)	{001}_Bss_ lattice vectors (Å)	Maximum linear and area misfit (Δ%)	Obliquity (°)	Notes
Case 1	1/3[441] = 12.855	2[010] = 13.860	+7.82		[120]_Bss_ twin axis
	4[010] = 19.958	[120] = 18.349	−8.79		
2D cell area (Å^2^) and multiplicity	161.733 (4×)	166.583 (2×)	+2.99	1.85	Opposite linear misfits

Case 2a	1/3[441] = 12.855	[110] = 13.883	+7.99		[120]_Bss_ twin axis
	1/3[4.14.1] = 21.540	3[010] = 20.780	−3.61		
2D cell area (Å^2^) and multiplicity	242.60 (6×)	249.87 (3×)	+2.99	4.34	Opposite linear misfits

Case 2b	−1/3[441] = 12.855	−[110] = 13.883	+7.99		[140]_Bss_
	6[010] = 29.938	[140] = 30.208	+0.90		
2D cell area (Å^2^) and multiplicity	242.60 (6×)	249.87 (3×)	+2.99	2.48	Coherent linear misfits

Case (3)	2/3[411] = 19.032	[120] = 18.349	−3.72		[210]_Bss_ twin axis
	5[010] = 24.948	[210] = 25.017	+0.276		
2D cell area (Å^2^) and multiplicity	404.33 (10×)	416.46 (5×)	+2.99	6.71	Opposite linear misfits

**Table 4:** 2D-LCs between {100}_Bss_ and {10.4}_Cc_ The {100} slices have to be of thickness *d*_200_, as required by the constraint 0*k*0 → *k* = 2*n*.

Ranking	{10.4}_Cc_ lattice vectors (Å)	{100}_Bss_ lattice vectors (Å)	Maximum linear and area misfit (Δ%)	Obliquity (°)	Notes
Case 1	1/3[441] = 12.855	2[010] = 13.86	+7.82		[021]_Bss_ twin axis
	4[010] = 19.958	[021] = 18.778	−6.28		
2D cell area (Å^2^) and multiplicity	161.733 (4×)	175.606 (2×)	+8.58	−3.38	Opposite linear misfits

Case 2	1/3[441] = 12.855	[001] = 12.67	−1.46		[032]_Bss_ twin axis
	1/3[8.13.2] = 22.061	3[010] = 20.79	−6.11		
2D cell area (Å^2^) and multiplicity	283.015 (7×)	263.409 (3×)	−7.44	3.65	Coherent linear misfits
