# Supplementary material for: Epitaxies of Ca sulfates on calcite. II. The main {010}, {001} and {100} forms of bassanite epi-deposited on the {10.4} substrate form of calcite
Source: J Appl Crystallogr. 2022 Sep 28;55(Pt 5):1289–96. doi: 10.1107/S1600576722008196 (PMC9533751; doi:10.1107/S1600576722008196)
Supplement: Supplementary file 1 [file j-55-01289-sup1.pdf]

JOURNAL OF APPLIED CRYSTALLOGRAPHY

Volume 55 (2022)

Supporting information for article:

Epitaxies of Ca-sulfates on calcite ($CaCO_3$). II. The main {010}, {001} and {100} forms of bassanite ($CaSO_4 \cdot 0.5H_2O$) epi-deposited on the substrate form of {10.4} calcite

Dino Aquilano, Marco Bruno, Stefano Ghignone, Linda Pastero and Andrea Cotellucci

Calcite is made by three supercells; each of them has multiplicity (2×) and is described by vectors and angles comprised between them, and that is: i) [020] = 9.979, ⅓×[$\bar{4}$11] = 9.516, δ = 121.62°; ii) -⅓×[45$\bar{1}$] = 9.516, -[020] = 9.979, δ = 121.62°, and -⅓×[$\bar{4}$11] = 9.516, ⅓×[45$\bar{1}$] = 9.516, ρ = 116.75°. The pseudo-hexagonal supercell with multiplicity (6×), occupies an area of 242.58 Å^2. The rectangular 2D-cell on the (10.4) face is drawn in Figure S1 (upper left side), while an example of one 2D-supercell has been drawn as well in the same figure (lower left side).

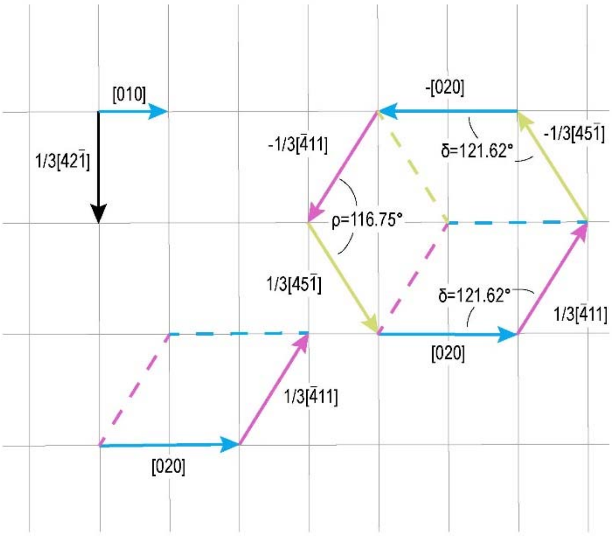

Figure S1. The pseudo-hexagonality of the cleaved {10.4} form of calcite. A (6×) supercell, (right side), made by three (2×) supercells, (left, lower side) is shown. The smallest 2D-cell of {10.4} is also drawn (upper left).

Table S1. Other 2D-LC between $\{001\}_{Bss}$ and $\{10.4\}_{Cc}$.

Ranking	$\{10.4\}_{Cc}$ lattice vectors (Å)	$\{001\}_{Bss}$ lattice vectors (Å)	Max. linear and area misfit (Δ%)	Obliquity (°)	Notes
case (2c)	$\frac{1}{3}$[$\bar{4}$41] = 12.8546<br>[43$\bar{1}$] = 24.816	[100] = 12.032<br>[$\bar{1}$30] = 24.013	-6.95<br>-3.34		No twin axis
2D cell area (Å)^2 and multiplicity	283.033 (7×)	249.87 (3×)	-13.27	0	Coherent linear misfits
case (4a)	[42$\bar{1}$] = 24.309<br>3[010] = 14.969	2[100] = 24.064<br>2[010] = 13.86	-1.02<br>-8.00		$[010]_{Bss}$ twin axis
2D cell area (Å)^2 and multiplicity	363.901 (9×)	333.166 (4×)	-9.22	0	Coherent linear misfits
case (4b)	$\frac{2}{3}$[$\bar{4}$11] = 19.032<br>5[010] = 24.948	[1$\bar{2}$0] = 18.349<br>[210] = 25.017	-3.72<br>+0.276		$[210]_{Bss}$ twin axis
2D cell area (Å)^2 and multiplicity	404.334 (10×)	416.458 (5×)	+2.99	6.71	Opposite linear misfits
